# Lymph Node Metastasis after a Soft Tissue Sarcoma of the Leg: A Case Report and a Review of the Literature

**DOI:** 10.1155/2013/930361

**Published:** 2013-02-06

**Authors:** S. D. Nelen, F. J. Vogelaar, F. Gilissen, J. C. Van der Linden, K. Bosscha

**Affiliations:** ^1^Department of Surgery, Jeroen Bosch Hospital, Henri Dunantstraat 1, 5223 GZ 's-Hertogenbosch, The Netherlands; ^2^Department of Pathology, Jeroen Bosch Hospital, Henri Dunantstraat 1, 5233 GZ 's-Hertogenbosch, The Netherlands

## Abstract

*Introduction*. Soft tissue sarcomas (STSs) represent 1 percent of all adult malignancies and sarcomas only rarely spread to the regional lymph nodes. *Case Presentation*. We present a case of a woman with a dermatofibrosarcoma protuberans and a sarcoma not therwise specified of the lower extremity. The patient had no distant metastasis during follow-up, but did develop a regional lymph nodemetastasis (RLNM) in the groin. We reviewed the literature about RLNM in STSs. *Discussion*. Reviewing the literature we see that within specific histological types RLNM occurs as often as distant metastasis. Furthermore RLNM occurs in over 10% for specific histological types and in 24% of all patients with a soft tissue sarcoma of the lower extremity. Except for radical lymphadenectomy with a 5-year survival rate of 46% there is no appropriate treatment. *Conclusion*. The risk for a RLNM in certain histological types and anatomical locations might transcend the risk for a distant lung metastasis.

## 1. Introduction

Soft tissue sarcomas (STSs) represent 1 percent of all adult malignancies and up to 6 percent of all childhood cancers [1–4]. In 2010, there were 10.520 cases of soft tissue sarcomas in the United States and 3920 patients died from this disease [5]. For dermatofibrosarcoma protuberans (DFSP) the annual incidence in the United States between 1973 and 2002 was 4.2 per 1.000.000 [6]. In the Netherlands the annual incidence of STS was 34 per 1.000.000 for men and 28 per 1.000.000 for women in 1997 [3].

The pattern of metastatic spread is usually haematogenous; lymphatic spread is very rare [7, 8]. We present a case of a patient with a inguinal lymph node metastasis 4 years after resection of a STS. A review of the literature will be described. 

## 2. Case Presentation

**Figure 1:**
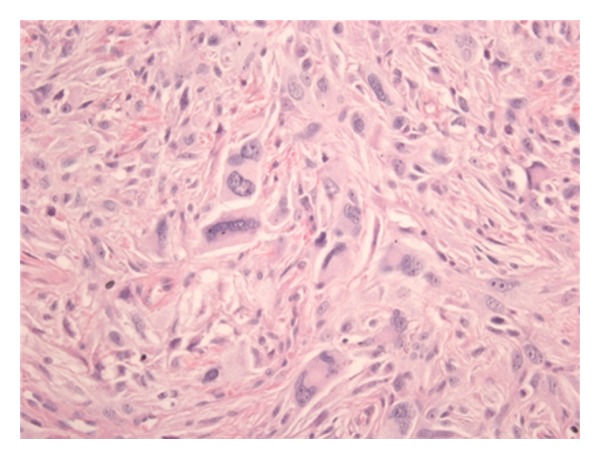
Microscopic view (400x) of the tumor of the right upper leg. The histopathological pattern, immunoprofile, and localization of this subcutaneous tumor favor the diagnosis of DFSP.

A 68-year-old Caucasian woman with hypertension and diabetes type 2 was treated in 2001 because of a mass of unknown histological origin of the right upper leg. She underwent a local resection of the tumor and pathological examination showed a DFSP. It was found to be a DFSP in histological research due to proliferation of atypical fusiform mesenchymal cells, with multinuclear giant cells which were sometimes arranged in rosettes (Figure 1). Moreover, the immunoprofile showed mainly positivity with vimentin (++), desmin (+), and CD-68 (+) which would mostly fit the diagnosis of a DFSP, a malignant fibrous histiocytoma was in the differential diagnosis. Due to tight surgical margins, a re-resection found place with margins of more than 2 centimetres.

**Figure 2:**
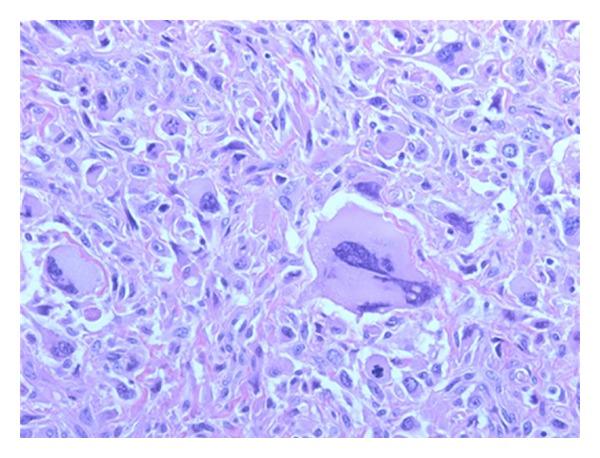
Microscopic view (400x) of the second tumor of the right upper leg. Based on the morphology, clinical history, and immunoprofile it was diagnosed to be a localization of a high grade sarcoma NOS.

During followup a new mass was discovered at the site of the scar 6 years later. Local surgical excision was performed and pathological examination showed a high grade sarcoma not otherwise specified (NOS), grade III. Histological research showed proliferation of a more cellular tumor process with more and bizarre mitotic figures (Figure 2). The tumor cells showed, compared to the primary tumor in 2001, less differentiation. The diagnosis of a NOS sarcoma was also based on the immunoprofile which was only positive for vimentin and the previously positive markers were considered negative (CD-68, desmin). After surgical excision the tumor was treated with radiotherapy. Ultrasound of the right groin showed no regional lymph node metastasis (RLNM) and there were no abnormalities seen at the chest X-ray.

**Figure 3:**
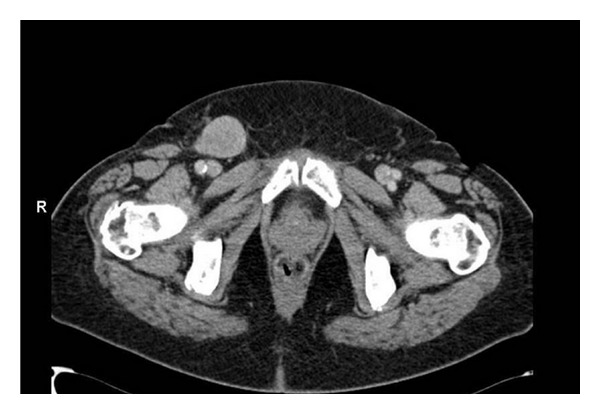
A 68-year-old Caucasian woman with a high grade sarcoma NOS presenting with a RLNM.

**Figure 4:**
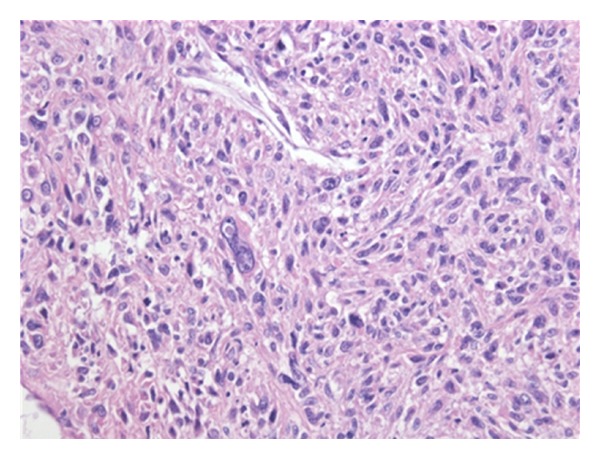
Microscopic view (400x) of the tumor located in a lymph node, diagnosed to be a localization of a high grade sarcoma NOS. It developed as a result of the differentiation of the primary tumor 10 years ago. This was concluded based on the morphology, clinical history, and immunoprofile (vimentin positive) of this tumor.

During further follow-up a groin mass was found 4 years later. CT examination revealed a large lymph node (Figure 3) without any other evidence of distant disease. An ultrasound-guided biopsy was performed and showed a metastases of the sarcoma. A superficial regional lymph node dissection was performed which showed one positive lymph node out of nine dissected and Cloquet's node negative. Pathological-anatomical study showed a metastasis of a high grade sarcoma NOS (Figure 4). Until now, further followup with chest X-ray and CT scan showed no abnormalities.

## 3. Discussion

### 3.1. Metastatic Pattern

In STS, distant metastases are relatively common, occurring in approximately 10% of patients. In patients with a sarcoma of the extremity, metastases will develop in up to 25% of patients [13, 14]. In contrary only 1% of patients with DFSP have distant metastases [6, 15]. Distant metastases occur especially in patients having had large, high grade tumors that are deep located to or nearby the fascia. Up to 83 percent of the distant metastases in STS occur in the lung [16, 17].

### 3.2. Lymph Node Metastases

Unlike distant metastases, regional lymph node metastases (RLNMs) in soft tissue sarcoma are rare. STS progress to regional lymph node metastases in 1.75%–5.9% [9–12]. Furthermore lymphatic spread is more frequently associated with certain histologic types, such as rhabdomyosarcoma, epitheloid sarcoma, clear cell sarcoma, or vascular sarcoma. Regional lymph node involvement is seen in over 10% of all patients [9–12]. Dermatofibrosarcoma protuberans and NOS-type sarcomas rarely spread to the regional lymph nodes (2%) (Table 1). Moreover RLNMs occur more often in patients having sarcomas of the lower extremities. The incidence of a RLNM in patients with a sarcoma of the lower extremity is reported up to 24% (Table 2) [9–12].

There is no research done in survival rates for patients with DFSP or NOS-type sarcomas and RLNMs. The median overall survival of patients with RLNM was 12.7 months, ranging from 0 to 40.7 months [9–12]. The 1-, 5-, and 10-year overall survival rates are 81.5%, 33.3%, and 20%. After the diagnosis of a regional lymph node the 1- and 5-year survival rates dropped to 55.5% and 12.8% [9–12]. The average time to develop RLNM is 27 months ranging from 1 month to 16 years after primary surgical resection [9–12]. For DFSP in general the 5-year survival rate was up to 99% [6, 15].

### 3.3. Therapy

To our knowledge there is no evidence of the single use of radiotherapy or chemotherapy in the treatment of STS with RLNM, nor is there any evidence for the specific treatment of primary NOS-type STS. For DFSP radiation therapy is only used in extremely large and recurrent tumors, and there is currently only a little role for chemotherapy [18].

In general for the treatment of a primary STS, radiation therapy combined with surgery is recommended only for intermediate and high grade malignancies [19, 20]. For low grade malignancies reexcision alone is favored over radiotherapy, and radiation therapy is not recommended with negative margins after surgery [19, 20]. The optimal timing of adjuvant radiation therapy in primary STS is not clear. In a prospective study randomizing for pre- or postoperative radiotherapy (RT) among 190 patients, there was a higher rate of acute wound complications with preoperative RT. Moreover there was a higher amount of patients with late complications, such as edema or fibrosis, for postoperative RT. Nevertheless there was no difference in survival rate between the two types of radiotherapy [21]. 

In general chemotherapy as part of the treatment of a primary STS in adults has not demonstrated an overall survival advantage [22, 23], nor is adjuvant chemotherapy considered to be a standard practice, in some studies even showing a worse 5-year survival rate [24, 25]. Furthermore, neo-adjuvant chemotherapy failed to show any benefit, awaiting the results of larger randomized trials [26, 27]. In contrast, hyperthermia has proven its efficacy in combination with neoadjuvant chemotherapy. In high-risk patients (>5 cm tumor size, grade 2 or 3, and/or deep to the fascia) it increases the benefit of chemotherapy alone [28–30]. 

The role of a sentinel lymph node biopsy(SLNB) for staging of patients with primary STS is unknown. In many cases, despite radical lymphadenectomy, patients with positive lymph node found with a SLNB procedure developed distant metastases; also a serious number of patients with positive lymph nodes remained disease-free. A multicenter trial would be necessary to determine the efficacy of SLNB [31–36].

At last we know that radical lymphadenectomy in case of RLNM remains the appropriate treatment so far [9]. Patients not treated with a radical lymphadenectomy had a shorter median survival than patients without a radical lymphadenectomy. Patients without appropriate treatment had a 5-year survival rate of 0% (median survival of 4.3 months) versus 46% (median survival of 16.3 months) after a radical excision of the regional lymph nodes [9]. 

## 4. Conclusion

To the best of our knowledge our case was the first description of a case of a DFSP followed by a high grade sarcoma NOS presenting with a RLNM. If we review the literature, there was a minor a priori chance of a RLNM in both types of STS. For DFSP the risk for a regional lymph node metastasis is as big as the risk for a distant metastasis; this is unknown for NOS-type soft tissue sarcoma. Nevertheless the standard of care in the Netherlands does not include a regular analysis of the regular nodes, but does include an X-ray for lung metastasis. 

Furthermore, we should urge caution in specific histological types and also in certain anatomical locations as the risk for an RLNM in rhabdomyosarcoma, epitheloid sarcoma, clear cell sarcoma, or a vascular sarcoma and also in STS of the lower extremities might transcend the risk for a distant lung metastasis.

According to the literature the best practice for a RLNM is radical surgery. There is yet no evidence on the therapy of RNLM with radiotherapy or chemotherapy but in primary tumors radiotherapy is proven effective. 

## Figures and Tables

**Table 1:** Four studies on the number of patients with a STS who develop RLNM.

Type of tumor	Number of patients	Number of RLNMs	Percentage
Synovial sarcoma			
Mazeron and Suit [9]	15	0	0%
Review Mazeron and Suit [9]	851	117	14%
Fong et al. [10]	145	2	1%
Daigeler et al. [11]	111	4	4%
Behranwala et al. [12]	171	7	4%

Total	1293	130	**10%**

Fibrosarcoma			
Mazeron and Suit [9]	45	0	0%
Review Mazeron and Suit [9]	215	54	25%
Fong et al. [10]	162	0	0%
Daigeler et al. [11]	45	1	2%
Behranwala et al. [12]	132	1	1%

Total	599	56	**9%**

Malignant fibrohistiocytoma			
Mazeron and Suit [9]	48	1	2%
Review Mazeron and Suit [9]	823	84	10%
Fong et al. [10]	316	8	3%
Behranwala et al. [12]	235	3	1%

Total	1422	96	**7%**

Neurofibrosarcoma			
Mazeron and Suit [9]	20	1	5%
Review Mazeron and Suit [9]	476	3	1%
Fong et al. [10]	96	2	2%
Daigeler et al. [11]	94	3	3%
Behranwala et al. [12]	95	4	4%

Total	781	13	**2%**

Liposarcoma			
Mazeron and Suit [9]	55	2	4%
Review Mazeron and Suit [9]	504	16	3%
Fong et al. [10]	403	3	1%
Daigeler et al. [11]	333	1	0%
Behranwala et al. [12]	340	3	1%

Total	1635	25	**2%**

Rhabdomyosarcoma			
Mazeron and Suit [9]	15	5	33%
Review Mazeron and Suit [9]	1354	201	15%
Fong et al. [10]	123	13	11%
Daigeler et al. [11]	50	3	6%
Behranwala et al. [12]	54	12	22%

Total	1596	234	**15%**

Leiomyosarcoma			
Mazeron and Suit [9]	30	1	3%
Review Mazeron and Suit [9]	524	21	4%
Fong et al. [10]	328	9	3%
Daigeler et al. [11]	167	1	1%
Behranwala et al. [12]	483	13	3%

Total	1532	45	**3%**

Vascular sarcoma			
Mazeron and Suit [9]	14	2	14%
Fong et al. [10]	37	5	14%
Daigeler et al. [11]	38	3	8%
Behranwala et al. [12]	46	5	11%

Total	135	15	**11%**

Epithelioid sarcoma			
Mazeron and Suit [9]	7	5	71%
Review Mazeron and Suit [9]	70	14	20%
Fong et al. [10]	12	2	17%
Daigeler et al. [11]	28	6	21%
Behranwala et al. [12]	27	5	19%

Total	144	32	**22%**

Clear cell			
Review Mazeron and Suit [9]	40	11	28%
Daigeler et al. [11]	14	3	21%
Behranwala et al. [12]	25	1	4%

Total	79	15	**19%**

NOS			
Mazeron and Suit [9]	42	2	5%
Fong et al. [10]	27	0	0%
Daigeler et al. [11]	268	2	1%
Behranwala et al. [12]	10	1	10%

Total	347	5	**1%**

Dermatofibrosarcoma Protuberans			
Daigeler et al. [11]	48	1	2%
Behranwala et al. [12]	43	1	2%

Total	91	2	**2%**

**Table 2:** Three studies on the number of patients with a STS who developed RLNM in various anatomical locations.

Anatomical location	Number of patients	Number of RLNMs	Percentage
Head and Neck			
Mazeron and Suit [9]	20	3	15%
Fong et al. [10]	45	5	11%
Behranwala et al. [12]	75	6	8%

Total	140	14	**10%**

Upper extremity			
Mazeron and Suit [9]	42	6	14%
Fong et al. [10]	47	7	15%
Behranwala et al. [12]	70	7	10%

Total	159	20	**13%**

Lower extremity			
Mazeron and Suit [9]	122	6	5%
Fong et al. [10]	46	19	41%
Behranwala et al. [12]	73	33	45%

Total	241	58	**24%**

Trunk			
Mazeron and Suit [9]	102	4	4%
Fong et al. [10]	500	5	1%
Behranwala et al. [12]	73	11	15%

Total	675	20	**3%**

Abdominal and Thoracic Viscera			
Mazeron and Suit [9]	23	0	0%
Fong et al. [10]	43	3	7%
Behranwala et al. [12]	73	11	15%

Total	139	14	**10%**
